# Surgery-related anxiety on geriatric patients undergoing total knee arthroplasty: a retrospective observational study

**DOI:** 10.1186/s12891-023-06252-w

**Published:** 2023-03-03

**Authors:** Kwang-Hwan Jung, Jang-Ho Park, Jae-Woo Ahn, Ki-Bong Park

**Affiliations:** 1 Department of Orthopedic Surgery, Ulsan University Hospital, University of Ulsan College of Medicine, 877 Bangeojinsunhwando-ro, Dong-gu, Ulsan, 44033 Republic of Korea; 2 Department of Psychiatry, Ulsan University Hospital, University of Ulsan College of Medicine, Ulsan, Republic of Korea

**Keywords:** Knee, Osteoarthritis, Total knee arthroplasty, State-anxiety

## Abstract

**Background:**

The prevalence of anxiety in patients undergoing total knee arthroplasty (TKA) and its association with postoperative functions are well known; however, the levels of anxiety or anxiety-related characteristics are unknown. This study aimed to investigate the prevalence of clinically significant state anxiety in geriatric patients undergoing TKA for osteoarthritis (OA) of the knee and to evaluate the anxiety-related characteristics experienced by these patients pre- and post-operatively.

**Methods:**

This retrospective observational study recruited patients who had undergone TKA for knee OA using general anesthesia between February 2020 and August 2021. The study participants were geriatric patients older than 65 years who had moderate or severe OA. We evaluated patient characteristics including age, sex, body mass index, smoking status, hypertension, diabetes, and cancer. We assessed their levels of anxiety status using the STAI-X which comprises 20-item scales. Clinically meaningful state anxiety was defined as a total score of 52 or higher. An independent Student’s t-test was used to determine differences of STAI score between subgroups in terms of patient characteristics. And patients were asked to complete questionnaires, which assessed four areas: (1) the main cause of anxiety; (2) the most helpful factor in overcoming anxiety before surgery; (3) the most helpful factor in reducing anxiety after surgery; and (4) the most anxious moment during the entire process.

**Results:**

The mean STAI score of patients who underwent TKA was 43.0 points and 16.4% of patients experienced clinically significant state anxiety. The current smoking status affect STAI score and the proportion of patients with clinically meaningful state anxiety. The most common cause of preoperative anxiety was the surgery itself. Overall, 38% of patients reported that they experienced the greatest level of anxiety when the surgeon had recommended TKA in the outpatient clinic. The trust in the medical staff before surgery and the surgeon’s explanations after surgery helped the most in reducing anxiety.

**Conclusions:**

One in six patients before TKA experience clinically meaningful state anxiety, and about 40% of patients experience anxiety from the time they are recommended for surgery. Patients tended to overcome anxiety before TKA through trust in the medical staff, and the surgeon’s explanations after surgery was found to be helpful in reducing anxiety.

## Background

Preoperative psychological anxiety is common in patients undergoing joint replacement surgery; reports have shown that approximately 25% of patients undergoing hip or knee arthroplasty have preoperative anxiety [1].

Anxiety is defined as an unpleasant emotional state consisting of expected emotional, cognitive, and behavioral changes in response to uncertainty about future threats [2]. Anxiety can be classified as trait-anxiety or state-anxiety. Trait anxiety refers to an individual’s susceptibility to anxiety, whereas state anxiety refers to the temporary anxiety arising from personal experiences with potential threats, and preoperative anxiety belongs to state anxiety [3]. Preoperative anxiety causes a variety of physiological and psychological reactions. Physiological responses included sweating, nausea, and a heightened sense of touch, smell, or hearing, along with an increase in pulse, blood pressure, and body temperature. Psychological responses include tension, nervousness, and aggression. In addition, preoperative anxiety is known to have a negative effect on patient satisfaction with surgery, and to affect the surgical success rate and the development of post-operative complications [1–3].

The overall prevalence of anxiety in patients undergoing total knee arthroplasty (TKA) and the association between anxiety and postoperative functional recovery are well known [4–7]. However, the prevalence of clinically meaningful state anxiety, patient characteristics that can affect the level of anxiety, and the main cause of anxiety, the most anxious period, and resolution factors are unknown. This study aimed to investigate the prevalence of clinically significant state anxiety in geriatric patients undergoing TKA for osteoarthritis (OA) of the knee and to evaluate the anxiety-related characteristics experienced by these patients pre- and post-operatively.

## Methods

### Study design

This retrospective observational study recruited geriatric patients who underwent general anesthesia and TKA for OA of the knee between February 2020 and August 2021.

### Participants

We included geriatric patients who were over 65 years and who had moderate or severe OA (Kellgren-Lawrence grade III or IV [8]) in this study. We excluded patients with rheumatoid arthritis, osteonecrosis, post-traumatic OA, a history of a psychiatric disorder, and those who did not consent to inclusion in this study. We evaluated patient characteristics including age, sex, body mass index, smoking status, hypertension, diabetes, and cancer (Table 1). We included 73 patients in the final sample. Of these, 83.6% (*n* = 61) were female, and the average age was 73.6 years (range, 65–85).

**Table 1:** Demographic for entire study population (*N* = 73)

Variables	N (%)
Sex	
Female: male	61 (83.6): 12 (16.4)
Body mass index	
Normal	11 (15.1)
Overweight	15 (20.5)
Obesity	47 (64.4)
Smoking status	
Nonsmoker	61 (83.6)
Former smoker	8 (11.0)
Current smoker	4 (5.4)
Hypertension	
+: -	44 (60.3): 29 (39.7)
Diabetes	
+: -	21 (28.8): 52 (71.2)
Cancer	
+: -	15 (20.5): 58 (79.5)
KL grade	
III: IV	48 (65.8): 25 (34.2)
	Mean (range)
Age (years, range)	73.6 (65–85)
Mean range of motion (°, range)	
Extension	−6.7 (−11.3–0)
Flexion	122 (86.3–138.4)
Mean varus deformity (°, range)	7.8 (1.4–14.3)

### Clinical pathway for TKA

We performed routine blood tests, chest radiography, electrocardiography, and pulmonary functional tests for all patients before admission to evaluate their functional abilities. General information about TKA surgery was first explained to all patients and their families in the outpatient clinic and we also provided explanations regarding the risks of general anesthesia and potential complications after TKA. A single surgeon in charge of adult joint arthroplasty performed all the surgeries and used Triathlon posterior-stabilized implant (Stryker, Kalamazoo, MI, USA) in all cases. The recovery process of all patients was accompanied by the same TKA rehabilitation protocols starting day 1 post-operatively. Rehabilitation focused on continuous passive mobilization at the patient’s bedside and included active range of motion exercises, calf pump exercises, straight leg raises, and quadriceps strengthening exercises. All patients walked with a walker and a knee brace to limit knee extension. All patients were discharged to their homes or nursing hospitals from our institution 2 weeks post-operatively. At the time of discharge, the physiologist explained home-based rehabilitation to the patients and informed them that they would follow-up with patients via a phone call 1 week after discharge (postoperative 3 weeks). The charge nurse explained the prescribed drugs and the date of follow-up at the outpatient clinic at postoperative 4 weeks.

### State-anxiety assessment and questionnaire

We measured anxiety status using the STAI-X type, which is the most used self-reported scale for subjective anxiety. It comprises 20-item scales and assesses levels of state anxiety [9]. Participants responded to each question on a Likert scale ranging from 1 to 4. The scores for each question were summed to calculate a total score ranging from 20 to 80. Higher scores correlated with a higher level of anxiety. Clinically meaningful state anxiety was defined as a total score of 52 or higher.

The authors designed a questionnaire to assess TKA-related anxiety based on previous studies of preoperative anxiety [10, 11]. The questionnaire consisted of four areas: (1) the main cause of anxiety, (2) the most helpful factor in overcoming anxiety before surgery, (3) the most helpful factor in reducing anxiety after surgery, and (4) the most anxious moment during the entire process. We distributed a questionnaire to all patients who were admitted for TKA and asked them to fill out the questionnaire immediately before discharge. One dedicated clinical research coordinator read the questionnaire to all patients the day before discharge (13 days after TKA) and helped them self-respond.

### Statistical analysis

An independent Student’s t-test was used to determine differences of STAI score between subgroups in terms of patient characteristics. All statistical analyses were performed using the IBM SPSS Statistics for Windows (version 24, IBM Corp., Armonk, NY, USA). A *p* value less than or equal to .05 was considered to indicate statistical significance.

### Ethics approval

This study was approved by the institutional review board of our institution, and written informed consent was obtained from all patients.

## Results

**Fig. 1:**
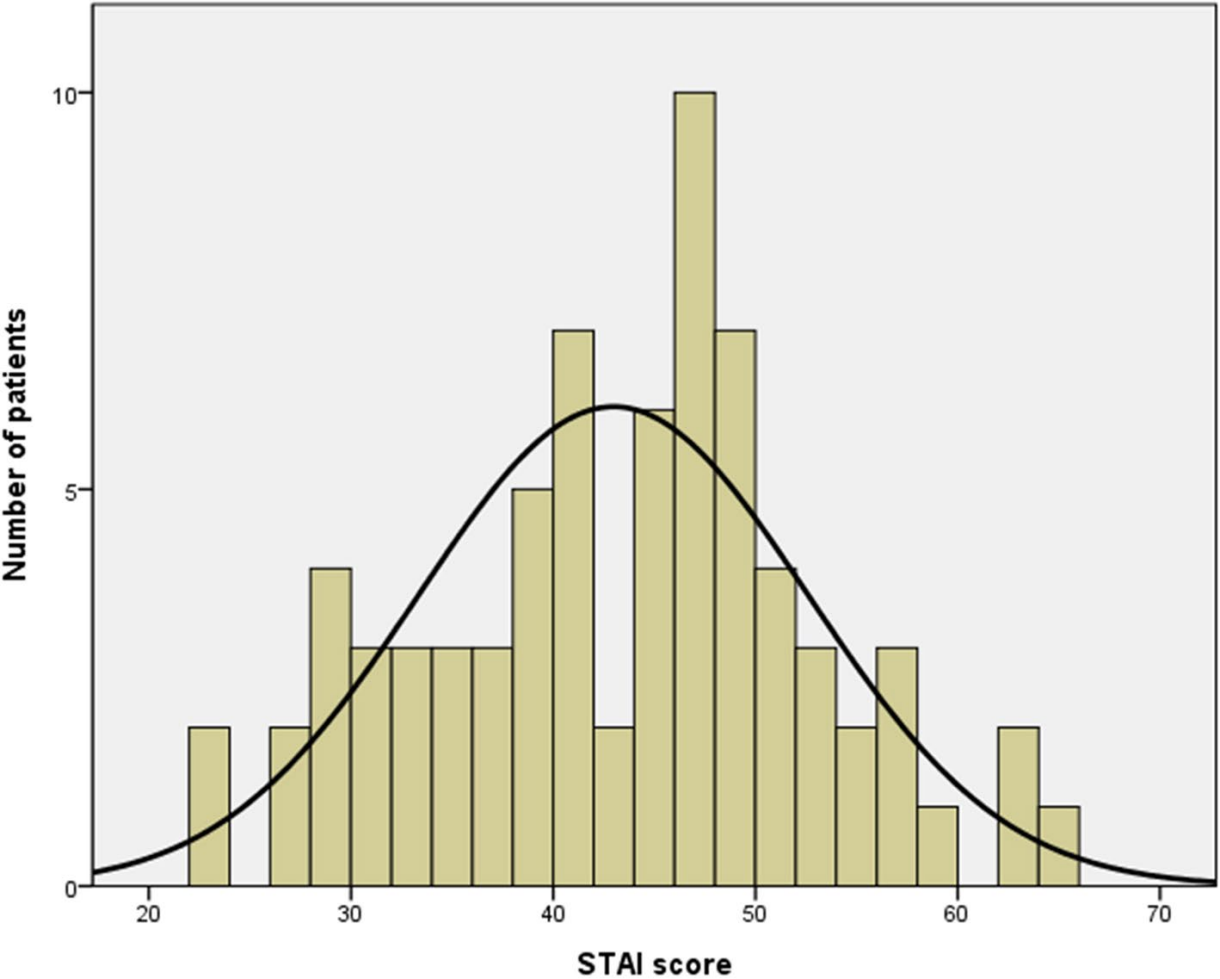
Levels of geriatric patient’s anxiety about total knee arthroplasty

The mean STAI score was 43.0 points (range, 23–65; Fig. 1). Twelve of the seventy-three patients (16.4%) showed a clinically meaningful state anxiety (STAI score ≥ 51).

**Fig. 2:**
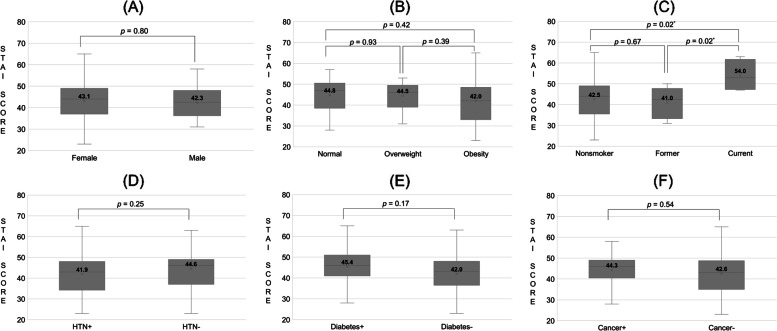
STAI score regarding patient characteristics. **A** sex, **B** body mass index, **C** smoking status, **D** hypertension, **E** diabetes, **F** cancer

Figure 2 shows STAI score regarding patient characteristics. Sex, body mass index, smoking status, diabetes, and hypertension did not show significant differences in mean STAI score. However, smoking status did affect STAI score (Fig. 2C). The mean STAI score of current smoker group (54.0) was significantly higher than that of the nonsmoker group (42.5) and former smoker group (41.0), and the *p*-values were 0.02 and 0.016, respectively.

Table 2 shows the proportion of patients with clinically meaningful state anxiety in subgroup in terms of patient characteristics. The proportion of patients with clinically meaningful state anxiety in current smoker group (50.0%) was significantly higher than that of nonsmoker group (16.4%, *p* = 0.03) and that of former smoker group (0%, *p* = 0.01).

**Table 2:** The proportion of patients with clinically meaningful state anxiety

	N (%)	*p* value
Sex (female: male)	11 (18.0): 1 (8.3)	0.41
Body mass index		
Normal^a,b^	2 (18.2)	^a^0.91
Overweight^a,c^	3 (20.0)	^b^0.79
Obesity^b,c^	7 (14.9)	^c^0.65
Smoking status		
Nonsmoker^d,e^	10 (16.4)	^d^0.22
Former smoker^d,f^	0 (0)	^e^0.01^*^
Current smoker^e,f^	2 (50.0)	^f^0.03^*^
HTN (+: -)	7 (15.9): 5 (17.2)	0.88
Diabetes (+: -)	5 (23.8): 7 (13.5)	0.29
Cancer (+: -)	1 (6.7): 11 (19.0)	0.26

^*^Statistically significant

^a,b,c,d,e,f^signified the *p* value according to the comparison between the two variables

Table 3 shows the main cause of anxiety regarding TKA. The most common cause of preoperative anxiety was the surgery itself (*n* = 31, 42.5%), this was followed by the risk of anesthesia (*n* = 18, 24.7%), postoperative rehabilitation (*n* = 10, 13.7%), postoperative pain (*n* = 9, 12.3%), and the separation from home and work (*n* = 5, 6.8%).

**Table 3:** Characteristics of patient anxiety about total knee arthroplasty

Characteristics of patient anxiety	N (%)
The main cause of anxiety	
Surgery itself	31 (42.5)
Risk of anesthesia	18 (24.7)
Postoperative rehabilitation	10 (13.7)
Postoperative pain	9 (12.3)
Separation from home and work	5 (6.8)
The most helpful factor in overcoming preoperative anxiety	
Trust in the medical staff	44 (60.3)
Family support	22 (30.1)
Religion	5 (6.8)
Others	2 (2.7)
The most helpful factor in reducing postoperative anxiety	
The surgeon’s explanation of the surgery	37 (50.7)
The fact that the surgery itself was completed	18 (24.7)
Improvement of symptoms	15 (20.5)
Others	3 (4.1)

The most helpful factor in overcoming preoperative anxiety was trust in medical staff (*n* = 44, 60.3%), followed by family support (*n* = 22, 30.1%), religion (*n* = 5, 6.8%), and other factors (*n* = 2, 2.7%).

The most helpful factor in reducing post-operative anxiety was the surgeon’s explanation of the surgery (*n* = 37, 50.7%), followed by the fact that the surgery itself was completed (*n* = 18, 24.7%), improvement in symptoms (*n* = 15, 20.5%), and others (*n* = 3, 4.1%).

**Fig. 3:**
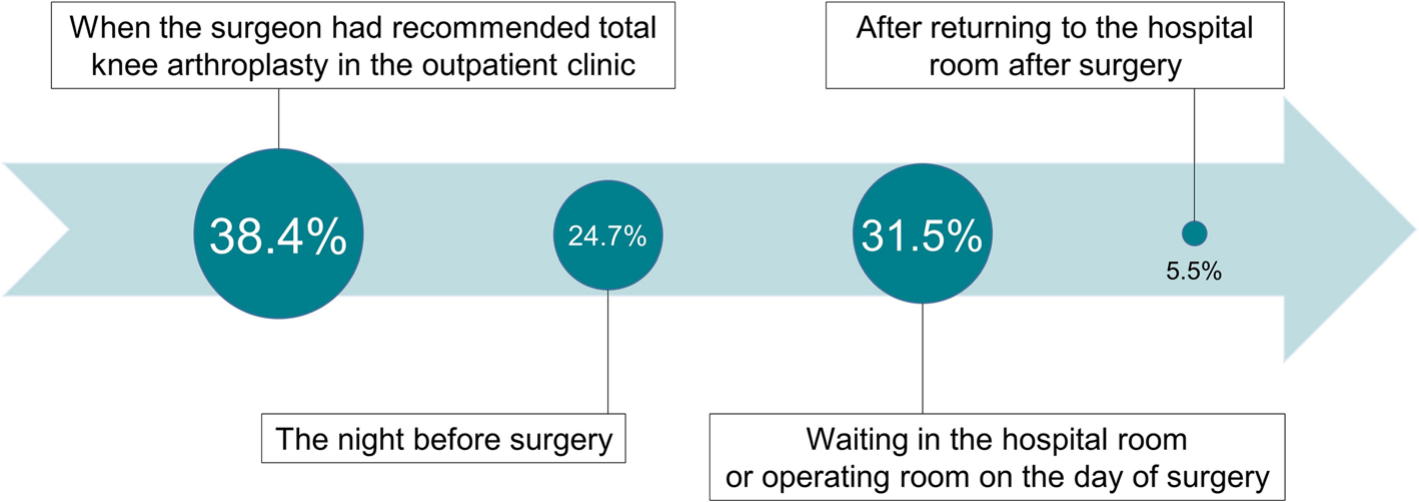
Most anxious moment during the whole process

The most anxious moment during the entire process was the moment when the surgeon had recommended TKA in the outpatient clinic (*n* = 28, 38.4%), followed by waiting in the hospital room or operating room on the day of surgery (*n* = 23, 31.5%), the night before surgery (*n* = 18, 24.7%), and after returning to the hospital room after surgery (*n* = 4, 5.5%) (Fig. 3).

## Discussion

This study showed that the mean STAI score of the geriatric patients who underwent TKA for OA of the knee was 43.0 points and 16.4% of patients experienced clinically significant state anxiety. The current smoking status affect STAI score and the proportion of patients with clinically meaningful state anxiety. The most common cause of preoperative anxiety was the surgery itself (42.5%). Overall, 38% of patients reported that they felt most anxious when the surgeon recommended TKA in the outpatient clinic. The trust in the medical staff before surgery and the surgeon’s explanations after surgery helped the most in reducing anxiety.

### STAI score

There are few studies which evaluated anxiety status using STAI in patients who underwent TKA and they reported that the average STAI score ranged from 29.6 to 64.5 [1, 12, 13]. In this study, STAI score ranged from 23 to 65.

### Clinically meaningful state anxiety

There seems to be no universal criteria to categorize anxiety levels; previous studies used various criteria to determine the levels of anxiety in patients who underwent TKA. Bian et al. [14] studied preoperative psychological distress in patients who underwent TKA and reported that 11.9% of patients had extremely severe or severe anxiety. Lippold et al. [15] studied the utility of symptoms of anxiety and depression in screening patients scheduled for TKA and reported that 15% of patients experienced heightened anxiety and/or depressive symptoms. This study used criteria which defined clinically meaningful state anxiety as a total STAI score of 52 or higher and found that 16.4% of geriatric patients who underwent TKA experienced clinically significant state anxiety.

### Preoperative anxiety about postoperative situations

In this study, 32.8% of the patients had anxiety about postoperative pain, rehabilitation after surgery, or being separate from home and work. Previous studies that analyzed the most important anxiety in patients who underwent TKA also reported that 24–58% of patients were most anxious about postoperative pain or rehabilitation [16–18]. Another study found that just over half (56.5%) of the participants were anxious about falls that may occur during rehabilitation after TKA [19]. We estimated that patients with OA of the knee, who suffered from pain and functional limitations for a long time due to chronic degenerative disease, were more interested in the difficulty of postoperative rehabilitation or returning to home and work compared to patients with acute hip fractures (10.7%) [11].

### Helpful factors to manage perioperative anxiety

Previous studies examined the factors that helped overcome surgery-related anxiety in patients with spinal disease or hip fracture; these studies reported that trust in the medical staff before surgery and the surgeon’s explanation after surgery were the most helpful measures in reducing anxiety [10, 11]. Similarly, in this study, majority of the geriatric patients stated that trust in the medical staff (60.3%) and the surgeon’s explanation of the surgery (50.7%) were the most helpful factors in overcoming pre- and post-operative anxiety.

### Most anxious period

In this study, 38% of the patients reported that they experienced the greatest level of anxiety when the surgeon recommended TKA in the outpatient clinic. Despite suffering from chronic knee pain for a long time, the treatment process in the outpatient clinic proceeded very quickly; that is, the patients received their updated OA diagnosis, were recommended joint replacement surgery, as the last treatment method, and were asked to decide the date of surgery within a few minutes. Therefore, we speculate that these situations in the outpatient clinic may be sufficient to induce anxiety in geriatric patients.

### Need for time-specific intervention for surgery-related anxiety

Reportedly, outpatient preoperative appointment with an anesthesiologist allows for patient education regarding different anesthetic options and counseling regarding anxiety related to anesthesia and surgery [20]. In this study, 24% of the total patients had preoperative anxiety surrounding the risk of anesthesia. As our institution did not have an outpatient preoperative appointment service, patients did not meet with an anesthesiologist preoperatively; however, orthopedic surgeons provided information about general anesthesia prior to surgery.

Ho et al. [1] introduced a patient-specific integrated education program for the TKA clinical pathway and compared pain intensity, anxiety scores, and functional scores between the intervention and control groups. They reported that STAI scores showed that patient anxiety status improved after intervention. We provided all patients with the same clinical pathway involved verbal preoperative education, preoperative rehabilitation, personalized rehabilitation during hospitalization, and physiologist-supervised home-based exercises after discharge.

Previous studies [21, 22] evaluated the effects of nursing interventions to reduce surgical anxiety. Although this study did not measure changes in the level of anxiety before and after surgery, we believe that the effect that routine surgical explanations during ward rounds has on patients cannot be ignored, as many patients overcame postoperative anxiety through explanations from medical staff. However, we think that there is a need for the medical staff to explain not only the information about the entire process of surgery but also the rehabilitation treatment process and provide an informative guide to patients regarding what to expect when they return to work or sport following TKA [23].

### Limitations

This study had some limitations. First, the sample size was small because only geriatric patients who underwent TKA for knee OA at a single institution were included. And the generalization of the findings of this study may be limited. However, this was beneficial in that this study was conducted in the same institution, provided the same medical staff, inpatient settings, surgical procedures, clinical pathways, and postoperative rehabilitation. Second, since the participants filled out the questionnaire the day before discharge, they must remind the experiences about surgery-related anxiety. Third, because we excluded patients with a psychiatric history, such as anxiety or depression, we could not analyze the prevalence of psychiatric history or newly developed anxiety in patients who underwent TKA. Fourth, this study showed that current smoking status could affect STAI score and clinically meaningful state anxiety. However, we did not analyze the level of smoking. Similarly, we did not analyze the effect of the level of previous smoking and the duration of smoking cessation in former smoker group. Finally, factors helpful in overcoming anxiety before and after surgery were investigated; however, it was impossible to specifically evaluate the extent to which anxiety decreased at each point.

## Conclusions

One in six patients before TKA experience clinically meaningful state anxiety, and about 40% of patients experience anxiety from the time they are recommended for surgery. Patients tended to overcome anxiety before TKA through trust in the medical staff, and the surgeon’s explanations after surgery was found to be helpful in reducing anxiety.

## Data Availability

The datasets used and/or analyzed during the current study are available from the corresponding author on reasonable request.

## Abbreviations

TKA: Total knee arthroplasty
OA: Osteoarthritis
